# Outcomes of operative and nonoperative treatment of 3- and 4-part proximal humeral fractures in elderly: a 10-year retrospective cohort study

**DOI:** 10.1007/s00068-017-0890-7

**Published:** 2017-12-28

**Authors:** Marieke E. Brouwer, Inge H. F. Reininga, Mostafa El Moumni, Klaus W. Wendt

**Affiliations:** Department of Trauma Surgery, University of Groningen, University Medical Center Groningen, Hanzeplein 1, PO Box 3.001, 9700RB Groningen, The Netherlands

**Keywords:** Proximal humeral fracture, Elderly, Nonoperative, Operative

## Abstract

**Purpose:**

Despite a rising incidence in proximal humeral fractures, there is still no evidence for the best treatment option, especially for elderly patients. The aim of this retrospective cohort study was to evaluate health-related quality of life (HRQoL), functional outcome, pain and social participation in elderly patients, after operative and nonoperative treatment of displaced 3- and 4-part proximal humeral fractures.

**Methods:**

150 patients aged ≥ 65, treated for a displaced 3- or 4-part proximal humeral fracture between 2004 and 2014, were invited to participate. Eventually 91 patients (61%) participated, of which 32 non-operatively treated patients were matched to 32 of the 59 operatively treated patients by propensity score matching. The EQ-5D, DASH, VAS for pain and WHODAS 2.0 Participation in Society domain were administered. Complications and reinterventions were registered.

**Results:**

No significant difference was found between the two treatment groups in HRQoL (*p* = 0.43), function (*p* = 0.78) and pain (*p* = 0.19). A trend toward better social participation in the operative group (*p* = 0.09) was found. More complications and reinterventions occurred in the operative group than the nonoperative group, with 9 versus 5 complications (*p* = 0.37) and 8 versus 2 reinterventions (*p* = 0.08).

**Conclusions:**

In this study, we found no evidence of a difference in HRQoL, functional outcome or pain 1–10 years after operative or nonoperative treatment in patients of 65 and older with a displaced 3- or 4-part humeral fracture. Operatively treated patients showed a trend toward better social participation but also higher reintervention rates.

## Introduction

Proximal humeral fractures are among the most common fractures in the elderly population [1]. Along with the increasing life expectancy of the Western population, the incidence of these fractures is rising rapidly, with osteoporosis as an important factor [2, 3]. Demographic research showed that proximal humeral fractures occur mostly in active persons aged 60 years and older [4]. Around 90% of these patients live independently at home and do their own shopping and housework. Hence, a proximal humeral fracture can potentially affect this independence and deteriorate the quality of life of the elderly.

Proximal humeral fractures can be classified as 1-, 2-, 3- or 4-part fractures according to the Neer classification, with 3- and 4-part fractures containing displaced fragments [5]. In case of a minimally or undisplaced fracture, the treatment is mostly nonoperative. For complex 3- and 4-part fractures both operative and nonoperative treatment are implemented in clinical practice [6–8]. Since the introduction of locking plates in the year 2000, operative treatment became a convenient option for elderly patients, as locking plates can also be used in osteoporotic bone [9, 10]. Consequently, operative treatment in elderly patients is performed more regularly than before the introduction of this technique [8]. Nevertheless, operative treatment is associated with a higher risk of complications related to the implant or the surgical procedure [11]. To date, research has not been able to identify evident and reliable differences in outcome between operative and nonoperative treatment [12–14]. This was supported by the latest Cochrane review [15].

Consensus is thus still lacking on the appropriate treatment for this type of fracture, especially for elderly patients. Previous studies focus mainly on the range of motion and functional and radiological outcome [6, 14, 16], paying little attention to functioning in daily life and social participation even though these outcomes are of the utmost importance to patients. According to the International Classification of Functioning, Disability and Health (ICF) of the World Health Organization (WHO), assessment of health and disability includes the effect of trauma not only on the affected body function or structure but also the assessment of limitations in activity and restrictions in social participation [17]. Hence the aim of this study was to assess the long-term outcome of operative and nonoperative treatment of displaced 3- and 4-part proximal humeral fractures in elderly patients in terms of impairments in body function or structure, limitations in activity and restrictions in social participation.

## Materials and methods

### Patients

**Fig. 1:**
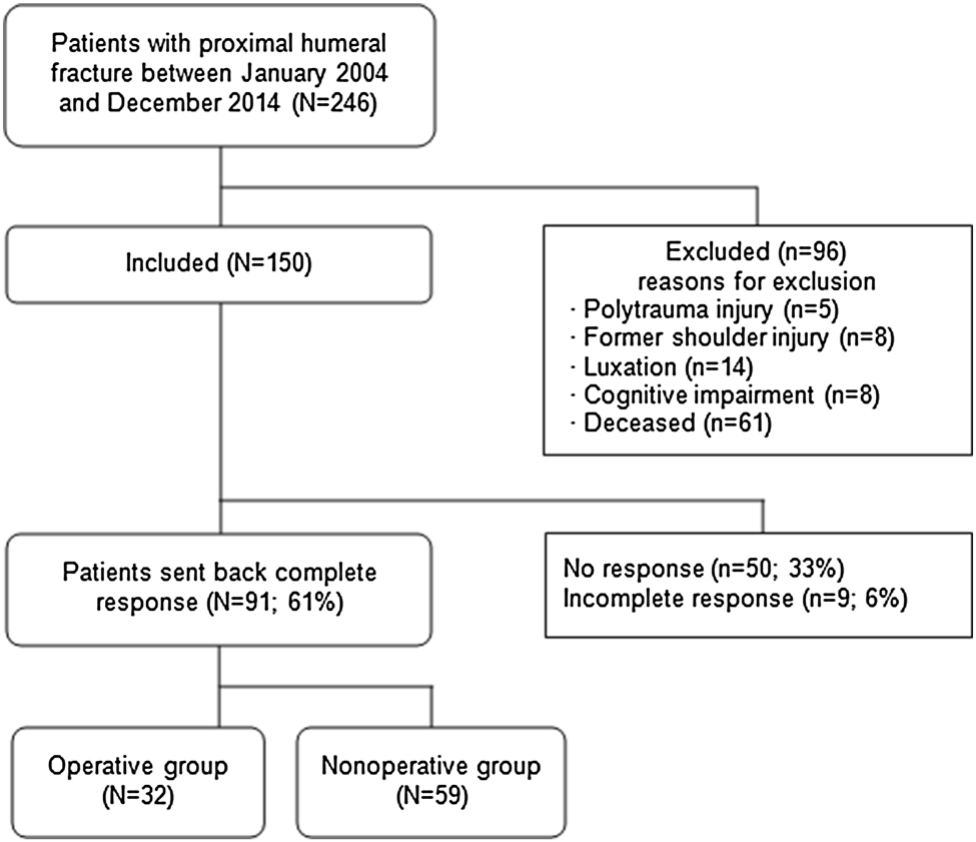
Flowchart of patient inclusion

The study design was a retrospective cohort study. Between January 2004 and December 2014, 246 patients were treated for a displaced 3- or 4-part proximal humerus fracture according to the Neer classification at the Department of Trauma Surgery of the University Medical Center Groningen, The Netherlands. All patients received either nonoperative or operative treatment. Operative treatment consisted of osteosynthesis by an intramedullary nail or locking plate, or hemiarthroplasty. Nonoperative treatment involved immobilization by a collar ‘n cuff and early physiotherapy after 1 week, as recommended by the Regional Trauma Protocol of Region West, The Netherlands [18]. The choice of treatment was executed by clinical judgment of the surgeon. In literature, different definitions of an “elderly” patient are used. In this study, patients aged 65 and older were included. Another inclusion criterion was low-energy trauma. Exclusion criteria were polytrauma, previous shoulder injury or surgery, shoulder dislocation and established dementia. After exclusion, a total of 150 patients were included for follow-up (Fig. 1). The local UMCG medical ethics committee judged the methods employed in this study and waived further need for approval (reference number METc 2015/181).

### Data collection

Medical records were checked for demographic information. Radiographs with an AP view and scapular Y view dating from the time of injury were viewed for fracture classification and affected side by two authors (MEB, KWW) and classified as a displaced 3-part or 4-part fracture type according to the Neer classification [5]. The fracture was defined as displaced when either the angulation between the two fragments exceeded 45° or the distance between the two fragments exceeded one centimeter. Treatment details and the occurrence of complications and reinterventions were retrieved from medical records. Complications directly related to the treatment or injury itself and reinterventions (secondary interventions) were recorded. Comorbidity was assessed by a 12-item comorbidity questionnaire developed by the National Institute for Public Health and the Environment that included the following conditions/diseases: migraine or severe headache, hypertension, lung disease, intestinal disorders, osteoarthritis, arthritis, severe back problems, diabetes mellitus, stroke, myocardial infarction, severe cardiovascular disease and cancer [19].

### Questionnaires

In September 2015, the patients received several questionnaires for follow-up by mail. Health-related quality of life (HRQoL) was assessed using the Euroqol-5D (EQ-5D) questionnaire [20]. The EQ-5D contains five dimensions: mobility, self-care, daily activities, pain/discomfort and anxiety/depression. The respondent indicates his/her state of health by selecting whether they have no problems, some problems or extreme problems in each dimension separately. This produces a total EQ-5D score ranging from 0 to 1, with 0 indicating the worst imaginable health and 1 indicating the best imaginable health. In this study the Dutch EQ-5D tariff was used [21].Physical functioning was assessed using the Disabilities of the Arm, Shoulder and Hand (DASH) questionnaire developed by the Institute for Work & Health and the American Academy of Orthopaedic Surgeons [22]. This questionnaire measures disability in daily activities and the severity of pain and other experienced symptoms of the upper extremity. DASH score ranges from 0 to 100, with 0 indicating no disability and 100 indicating full disability. Additionally, a visual analog scale (VAS) was used to quantify shoulder pain [23]. VAS scores range from 0 to 100, with 0 indicating no pain and 100 indicating the worst imaginable pain.

To assess social participation, the Participation in Society domain of the WHODAS 2.0 was used [24]. The WHODAS 2.0 is an instrument developed by the World Health Organization (WHO) to assess the limitations and restrictions to participation that an individual experiences because of their health problems, independently from a medical diagnosis. The Participation in Society domain consists of eight questions. Respondents are asked to indicate the amount of impediment to participation they experienced in the last 30 days by selecting ‘none, mild, moderate, severe, or extreme’. The final score ranges from 0 to 100, with 0 indicating no disability and 100 indicating full disability.

### Statistical analysis

Patients in the operative group were matched to patients from the nonoperative group by propensity score matching to reduce the effect of “treatment by indication”. This is an adjunct disadvantage of retrospective cohort studies in which the treatment is selected by clinical judgment of the surgeon and not assigned randomly as in a randomized controlled trial. With this method, a propensity score is calculated using logistic regression. Propensity score can be defined as the probability that a patient received a particular treatment given the included covariates [25]. The variables used for propensity score matching were gender, age at injury, educational level, comorbidity, fracture type and affected side. The matching procedure was performed using the program R and package MatchIt [26, 27]. Both balance statistics and plots were used to check the balance between the operatively- and non-operatively-treated patients as provided by the MatchIt package [17].

Data were analyzed using SPSS version 22.0 for Windows (IBM Corporation, Armonk, NY) [28]. Education, comorbidity, occurrence of complications and occurrence of reinterventions were coded as dichotomous variables. Educational level was divided into “lower” and “higher”. “Lower” meaning the patient had no education, finished elementary school, secondary school or intermediate vocational education college, and “higher”, meaning the patient received a Bachelor’s degree or higher. Patient comorbidity was coded as “no” or “yes”, with “no” meaning the patient was not affected by any of the conditions on the 12-item comorbidity questionnaire in the past 6 months, and “yes” meaning the patient was affected by one or several conditions on this list in the past 6 months. The mean number of comorbidities was also reported.

Baseline characteristics of non-responders and responders were compared using an independent T-test for continuous variables and Fisher’s exact test for dichotomous variables to check for response bias. Baseline characteristics of the study cohort before and after propensity score matching were compared in the same manner, to assure comparability of the two treatment groups. Normal distribution of the outcome measures was checked using histograms and Q–Q plots. Means and standard deviations of the outcome measures were reported if normally distributed, medians and ranges if not normally distributed.

Linear regression analysis was performed using the matched data to assess the effect of treatment modality (operative versus nonoperative) on EQ-5D, DASH, VAS for pain and WHODAS scores. Effect modification was examined for gender, age at the time of injury, educational level, comorbidity, fracture type, affected shoulder and follow-up time. Effect modification was assumed to be significant at a *p* value of 0.05. The variables of gender, current age, educational level, fracture type, affected side, comorbidity and follow-up time were checked for confounding by a forward-stepwise selection procedure, adding a variable as confounder to the model if this contributed by a change > 10% to the original regression coefficient. The difference in outcome between the two treatment groups could be defined by the final regression coefficient of the variable treatment. The occurrence of complications and reinterventions in the operative and nonoperative group were compared using Fisher’s exact test. Results were considered statistically significant at *p* < 0.05.

## Results

Of the 246 patients treated for a displaced 3- or 4-part proximal humeral fracture at UMCG between January 2004 and December 2014, 35 patients did not meet the inclusion criteria. Additionally, 61 patients died from causes unrelated to the treatment or injury (Fig. 1). Of the 150 patients included, 91 (61%) completed the questionnaires. Of these patients, 59 (65%) underwent nonoperative treatment and 32 (35%) operative treatment. Mean follow-up was 58 months (range 10–131 months). Non-response analysis showed that non-responders were significantly older and had a significantly longer follow-up (Table 1).

**Table 1:** Demographic characteristics of responders and non-responders

Characteristic^a^	Responders	Non-responders	*p* value
Patients	91 (61)	59 (39)	
Age at injury (years)	73 ± 6.0	76 ± 7.4	0.002
Gender			
Female	76 (84)	45 (76)	0.30
Male	15 (16)	14 (24)	
Fracture classification			
3-part	68 (75)	46 (78)	0.70
4-part	23 (25)	13 (22)	
Affected shoulder			
Right	39(43)	27 (46)	0.74
Left	52 (57)	32 (54)	
Follow-up time (months)	52 ± 31.7	67 ± 33.7	0.005

^a^Data are presented as *N* (%) or, mean ± standard deviation

### Patient characteristics

Before propensity score matching, the nonoperative group consisted of 9 males (15%) and 50 females (85%), mean age 77 ± 7.0, with 50 (85%) 3-part and nine (15%) 4-part fractures. Mean follow-up was 47 ± 30.3 months. The operative group consisted of six males (19%) and 26 females (81%), mean age 77 ± 5.8, with 18 (56%) 3-part and 14 (44%) 4-part fractures. Mean follow-up was 61 ± 32.8 months (Table 2). Of the 32 operatively treated patients, 23 underwent osteosynthesis (a locking plate in 12 patients and an intramedullary nail in 11 patients) and nine underwent hemiarthroplasty. Before matching there was a statistically significant difference between the two groups for fracture type and follow-up time (*p* = 0.005 and *p* = 0.05, respectively). After propensity score matching, 32 of the non-operatively treated patients were matched to the 32 operatively treated patients. Matching was successfully performed, leaving no significant differences between the operative and nonoperative groups for the variables. After matching the nonoperative group consisted of five males (16%) and 27 females (84%), mean age 76 ± 6.2, with 23 (72%) 3-part fractures and nine (28%) 4-part fractures. Mean follow-up was 46 ± 31.6 months (Table 2).

**Table 2:** Demographic characteristics of the study cohort before and after propensity score matching

Characteristic^b^	Before PSM^a^			After PSM		
	Nonoperative (*n* = 59)	Operative (*n* = 32)	*p* value	Nonoperative (*n* = 32)	Operative (*n* = 32)	*p* value
Current age (years)	77 ± 7.0	77 ± 5.8	0.84	76 ± 6.2	77 ± 5.8	0.59
Age at injury (years)	72 ± 6.6	72 ± 4.8	0.60	72 ± 5.9	72 ± 4.8	0.91
Gender						
Female	50 (85)	26 (81)	0.77	27 (84)	26 (81)	1.000
Male	9 (15)	6 (19)		5 (16)	6 (19)	
Level of education						
Low	48 (81)	28 (87)	0.56	30 (94)	28 (87)	0.67
High	11 (19)	4 (14)		2 (6)	4 (14)	
Fracture classification						
3-part	50 (85)	18 (56)	0.005	23 (72)	18 (56)	0.30
4-part	9 (15)	14 (44)		9 (28)	14 (44)	
Affected shoulder						
Right	26 (44)	13 (41)		13 (41)	13 (41)	1.000
Left	33 (56)	19 (59)	0.83	19 (59)	19 (59)	
Comorbidity present						
Yes	47 (78)	27 (84)		4 (13)	5 (16)	1.000
No	12 (20)	5 (16)	0.78	28 (87)	27 (84)	
Mean number of comorbidities	1.9 ± 1.6	1.8 ± 1.3	0.95	2.4 ± 1.7	1.8 ± 1.3	0.15
Follow-up time (months)	47 ± 30.3	61 ± 32.8	0.051	46 ± 31.6	61 ± 32.8	0.086

^a^*PSM* Propensity Score Matching

^b^Data are presented as *N* (%) or, mean ± standard deviation

### Follow-up

Since all patients included for matching returned a complete response to the questionnaires, there was no missing data. The DASH, VAS and EQ-5D were normally distributed. WHODAS score was not normally distributed. Means and standard deviations and medians and ranges are summarized in Table 3. None of the variables showed significant effect modification. Table 4 displays the differences in EQ-5D, DASH, VAS and WHODAS between operatively and non-operatively treated patients after correction for confounding variables. No statistically significant difference in EQ-5D score was found between the operative and nonoperative groups (*p* = 0.43). No significant differences were found for DASH (*p* = 0.78) and VAS for pain (*p* = 0.19) either. A trend towards lower WHODAS scores in the operative group with a difference of 10.8 points (*p* = 0.09) was observed (Table 4).

**Table 3:** Description of the EQ-5D, DASH, VAS for pain and WHODAS scores

	Total group	Nonoperative	Operative
EQ-5D^a^	0.72 ± 0.24	0.70 ± 0.28	0.74 ± 0.21
DASH^a^	31.9 ± 24.9	32.8 ± 26.8	31.0 ± 23.2
VAS^a^	2.6 ± 2.5	3.0 ± 2.6	2.3 ± 2.3
WHODAS^b^	18.8 (0–100)	21.9 (0–100)	17.2 (0–56.3)

^a^Data presented as mean ± standard deviation

^b^Data presented as median (range)

**Table 4:** Results of linear regression analysis

	Variable	Coefficient	95% CI	*p*
EQ-5D	Treatment	0.05^a^	− 0.08 to 0.17	0.43
	Current age	− 0.02	− 0.03 to − 0.004	0.008
	Follow-up time	0.001	− 0.001 to 0.003	0.51
	Fracture type^b^	− 0.06	− 0.18 to 0.07	0.38
DASH	Treatment	− 1.8^a^	− 14.7 to 11.1	0.78
	Current age	1.2	0.05 to 2.4	0.04
	Fracture type	4.2	− 9.0 to 17.4	0.53
	Follow-up time	− 0.1	− 0.30 to 0.13	0.44
	Gender	− 8.0	− 25.9 to 9.9	0.38
	Comorbidity^c^	6.6	− 11.2 to 24.5	0.46
VAS	Treatment	− 0.8^a^	− 2.1 to 0.42	0.19
	Fracture type	0.6	− 0.75 to 1.9	0.39
WHODAS	Treatment	− 10.8^a^	− 23.2 to 1.5	0.09

Reference groups: ^a^nonoperative group, ^b^3-part fracture, ^c^no comorbidity

### Complications and reinterventions

Table 5 displays the complications and reinterventions. In the operative group, nine patients (28%) experienced a complication related to shoulder fracture or its treatment within 2 years of injury, versus five patients (16%) in the nonoperative group. This difference was not statistically significant (*p* = 0.37). Reported complications in the nonoperative group were restricted movement, nonunion, avascular head necrosis and persistent pain. Reported complications in the operative group were discomfort from the osteosynthetic material, restricted movement, failure of the osteosynthetic material, wound infection and persistent pain. The occurrence of reintervention showed a trend towards more reinterventions in the operative group, where eight patients (33%) required removal of osteosynthesis material, compared to the nonoperative group, where two patients (6%) required hemiarthroplasty (*p* = 0.08). One patient from the operative group required hemiarthroplasty after removing the osteosynthesis material.

**Table 5:** Data on patient complications and reintervention

Group^a^	Material^b^	Gender/age	Fr. type	Complication	Reintervention
NO		M/82	3-part	Restricted movement	
NO		F/72	4-part	Restricted movement	Hemiarthroplasty
NO		F/66	3-part	Persistent pain	
NO		F/66	3-part	AVN	
NO		M/67	3-part	Non-union	Hemiarthroplasty
O	LP	F/66	4-part	Wound infection	Extraction of plate + Hemiarthroplasty
O	IN	F/65	3-part	Discomfort of osteosynthesis material	Extraction of nail, release
O	IN	F/68	3-part	Restricted movement	Extraction of screw
O	IN	F/68	3-part	Failure of osteosynthesis material	Extraction of nail, release
O	IN	F/69	3-part	Failure of osteosynthesis material	Extraction of nail, release
O	LP	F/71	3-part	Persistent pain	Extraction of plate
O	HA	F/71	4-part	Wound infection	
O	LP	F/72	3-part	Discomfort of osteosynthesis material	Extraction of plate screw
O	IN	F/71	4-part	Discomfort of osteosynthesis material	Extraction of screw

^a^*NO* nonoperative, *O* operative

^*b*^*LP* locking plate, *IN* intramedullary nail, *HA* hemiarthroplasty

## Discussion

This study presents long-term outcomes of displaced 3- and 4-part proximal humeral fractures in a multidimensional way, focusing primarily on HRQoL outcome and additionally on physical functioning, pain, social participation, complications and reinterventions. As it is difficult to realize a randomized controlled trial about this type of fracture, retrospective studies dominate the field. Using propensity score matching, this study tried to minimize selection bias caused by “treatment by indication”. This study found no significant differences in outcome between operatively and non-operatively treated patients regarding HRQoL, physical functioning or pain. Operatively treated patients showed a trend toward better social participation. No significant difference in complication and reintervention occurrence between the two groups was found, although a trend was seen toward more reinterventions after operative treatment.

This study focused on a more specific group of patients, namely patients of 65 and older with a displaced 3- or 4-part proximal humeral fracture. Many previous studies also included younger patients, inclusion starting from 18 years of age, and 2-part fractures [6, 29]. Some studies chose to include only 3-part or only 4-part fractures but not both [12, 30]. This should not be overlooked when comparing results. Still, demographic characteristics of the patients in this study were representative of the general population of patients with proximal humeral fractures, as shown by several epidemiological studies [2, 3, 31].

No statistically significant difference in HRQoL between operatively and non-operatively treated patients was found. This finding is in accordance with several previous studies [12, 13, 29]. The average EQ-5D score found in this study is considerably lower than the EQ-5D reference value for the general Dutch population aged over 65 [32], which supports the claim that a proximal humeral fracture in elderly patients/patients over 65 is related to a diminished quality of life.

Both operatively and non-operatively treated patients showed a mild limitation in physical functioning, demonstrated by a higher mean DASH score, compared to the general Norwegian older population (mean 18, 22, 36 in women and 11, 13, 22 in men of 60–69, 70–79 and 80 +, respectively); this is comparable to the general Dutch older population [33, 34]. This finding supports the claim that this type of injury influences physical functioning in the long term. The results in functional outcome of this study correspond with functional outcome measured in several previous studies [12, 30]. Some studies do report better functional outcome, probably due to the inclusion of patients with 2-part fractures and patients of 18 years and older, resulting in a lower mean age of the study cohort compared to this study [35–37]. This contributes to the assumption that older patients with 3- and 4-part fractures should be studied separately from younger patients when it comes to treatment and outcome. None of the studies mentioned above was able to find significant differences in physical functioning between operatively and non-operatively treated patients.

The low-to-moderate pain level found in this study corresponds with results from previous studies [30, 38–40]. Pain levels favored operative treatment with a difference of 0.8 on the VAS, which is very similar to the 1.0 points lower score after hemiarthroplasty reported by Olerud et al. [30]. Lack of power could be the reason that both studies failed to show statistical significance.

To our knowledge, social participation has not been used before as outcome measure after a proximal humeral fracture. According to the ICF model, the assessment of health and disability should also comprise the assessment of social participation. This study found a trend toward better social participation after operative treatment, meaning operatively treated patients reported experiencing fewer problems with participation in society caused by their health condition than non-operatively treated patients. The inability to present a statistically significant difference could be caused by the lack of statistical power of a small patient population. Further research with larger study cohorts and the use of social participation as outcome measure is desirable.

Complications and reinterventions occurred slightly more often after operative treatment, though this was statistically not significant. This failing of reaching statistical significance might be due to the small sample size of the study cohort. In this study, 33% of the operatively treated patients required surgery after primary treatment versus 6% of the non-operatively treated patients. This finding is not surprising, as many reinterventions consisted of removing the osteosynthetic material due to discomfort, a complication related to the osteosynthesis material itself. The high risk of reintervention after osteosynthesis has been described in the literature before and might be reduced when improving the surgical technique [41]. Two patients from the nonoperative group eventually underwent hemiarthroplasty as a reintervention. Because this study comprises an intention-to-treat analysis, these patients were left included in the nonoperative group. This study reported all complications registered in medical records, i.e., patient-reported complications that led to pain, discomfort and reintervention. Consequently, asymptomatic complications, like some cases of avascular necrosis of the humeral head (AVN) [42], were not reported but are considered of minor significance to patient well-being and satisfaction.

The retrospective design of this cohort study has some limitations. First, only 61% of contacted patients responded to the questionnaires, which might have led to response bias. Compared to the non-response group, a higher percentage of women was included in the study. However, the male/female ratio of the study population is in accordance with the overall population of elderly patients/patients over 65 with a proximal humeral fracture [3, 43]. Second, choice of treatment was executed by clinical judgment of the surgeon and not assigned randomly. We used propensity score matching to minimize dependency between the treatment variable and the other covariates. Third, since the objective of this study was to compare the outcomes of nonoperative treatment with those of operative treatment, this study did not distinguish between surgical techniques, such as open reduction and internal fixation and hemiarthroplasty; it, however, is a reflection of general clinical practice, where the type of surgical procedure is based on the clinical judgment of the surgeon. Additionally, locking plates, intramedullary nails and hemiarthroplasty are the most commonly used surgical techniques in current practice [8, 44].

Lastly, since it is hard to realize large patient numbers in proximal humeral fracture studies, this study included a small number of patients. Despite the small number of patients, this study has demonstrated the importance of measuring outcome on multiple levels of functioning and disability, including the measurement of social participation. Also, this study emphasizes that operative treatment in elderly patients/patients over 65 should be considered carefully, as it is accompanied by a high risk of reintervention. However, this study cannot be conclusive on the best treatment for these patients yet. More research with larger study cohorts is desirable.

## Conclusion

This study revealed that surgical intervention of displaced 3- and 4-part proximal humerus fractures did not yield significantly better outcomes than nonoperative treatment in patients over the age of 65 regarding HRQoL, function, pain, social participation, complications and reintervention. A trend toward better social participation, but also more reinterventions, after operative treatment was found. This study stresses the importance of weighing the possible advantages of operative treatment against the high risk of reintervention, particularly when treating frail elderly patients/patients over 65. We emphasize the need for more research focusing on this specific patient group and the surplus value of measuring all three levels of the ICF model when comparing treatments.
